# Phosphaturic mesenchymal tumor-induced bilateral osteomalacia femoral neck fractures: a case report

**DOI:** 10.3389/fendo.2024.1373794

**Published:** 2024-04-16

**Authors:** Yifan Zhang, Mingwei Hu, Cuicui Guo, Xue Yang, Shuai Xiang, Hao Xu

**Affiliations:** ^1^ Department of Joint Surgery, The Affiliated Hospital of Qingdao University, Shandong, China; ^2^ Department of Sports Medicine, The Affiliated Hospital of Qingdao University, Shandong, China; ^3^ Department of Operation Room, The Affiliated Hospital of Qingdao University, Shandong, China

**Keywords:** phosphaturic mesenchymal tumor, tumor-induced osteomalacia, femoral neck fracture, thoracic spine, hypophosphoruria, total hip arthroplasty

## Abstract

Phosphaturic mesenchymal tumors (PMT) are rare and distinctive tumors that typically result in paraneoplastic syndrome known as tumor-induced osteomalacia (TIO). We report a case of bilateral osteoporotic femoral neck fracture caused by PMT. PMT was surgically resected, followed by sequential treatment of bilateral femoral neck fractures with total hip arthroplasty (THA). A 49-year-old perimenopausal woman experienced consistent bone pain with limb weakness persisting for over 2 years. Initially, she was diagnosed with early osteonecrosis of the femoral head and received nonsurgical treatment. However, from 2020 to 2022, her pain extended to the bilateral shoulders and knees with increased intensity. She had no positive family history or any other genetic diseases, and her menstrual cycles were regular. Physical examination revealed tenderness at the midpoints of the bilateral groin and restricted bilateral hip range of motion, with grade 3/5 muscle strength in both lower extremities. Laboratory findings revealed moderate anemia (hemoglobin 66 g/L), leukopenia (2.70 × 10^9^/L), neutropenia (1.28 × 10^9^/L), hypophosphatemia (0.36 mmol/L), high alkaline phosphatase activity (308.00 U/L), and normal serum calcium (2.22 mmol/L). After surgery, additional examinations were performed to explore the cause of hypophosphatemic osteomalacia. After definitive diagnosis, the patient underwent tumor resection via T11 laminectomy on August 6, 2022. Six months after the second THA, the patient regained normal gait with satisfactory hip movement function without recurrence of PMT-associated osteomalacia or prosthesis loosening. By providing detailed clinical data and a diagnostic and treatment approach, we aimed to improve the clinical understanding of femoral neck fractures caused by TIO.

## 1 Introduction

Phosphaturic mesenchymal tumors (PMT) are extremely rare, with only approximately 450 cases being reported to date in the literature, the vast majority of which are benign (1). PMT is often diagnosed in middle-aged adults; however, it has also been observed in children and in older patients. PMT is characterized by elevated levels of fibroblast growth factor‐23 (FGF23), which is a phosphaturic hormone produced by the bone that reduces serum phosphate levels by suppressing proximal tubular phosphate re-absorption (2). FGF23 can also influence systemic vitamin D activity by suppressing the renal expression of 1α-hydroxylase, resulting in a decrease in calcitriol production (3). Hypophosphatemia and tumor-induced osteomalacia (TIO) are the main consequences of these processes. Recently, PMT has been recognized as one of the leading causes of TIO (1). A study by Lee et al. (4) showed that the fusion of FN1 (which encodes fibronectin 1) and FGFR1 (which encodes the fibroblast growth factor receptor 1) genes is expressed in half of PMT (41%) cases. This result suggests that the FN1-FGFR1 fusion gene plays a critical role in FGFR1 signaling pathways, leading to overexpression of FGF23 and tumor growth.

Here, we present a case of a woman with bilateral osteoporotic femoral neck fractures. PMT was discovered during the treatment of the fractures. The patient underwent PMT resection and total hip arthroplasty (THA) sequentially. Here, we describe the clinical manifestations, diagnostic process, treatment and prognosis of this case to aid in the clinical diagnosis and treatment of femoral neck fractures caused by TIO.

## 2 Case description

**Figure 1:**
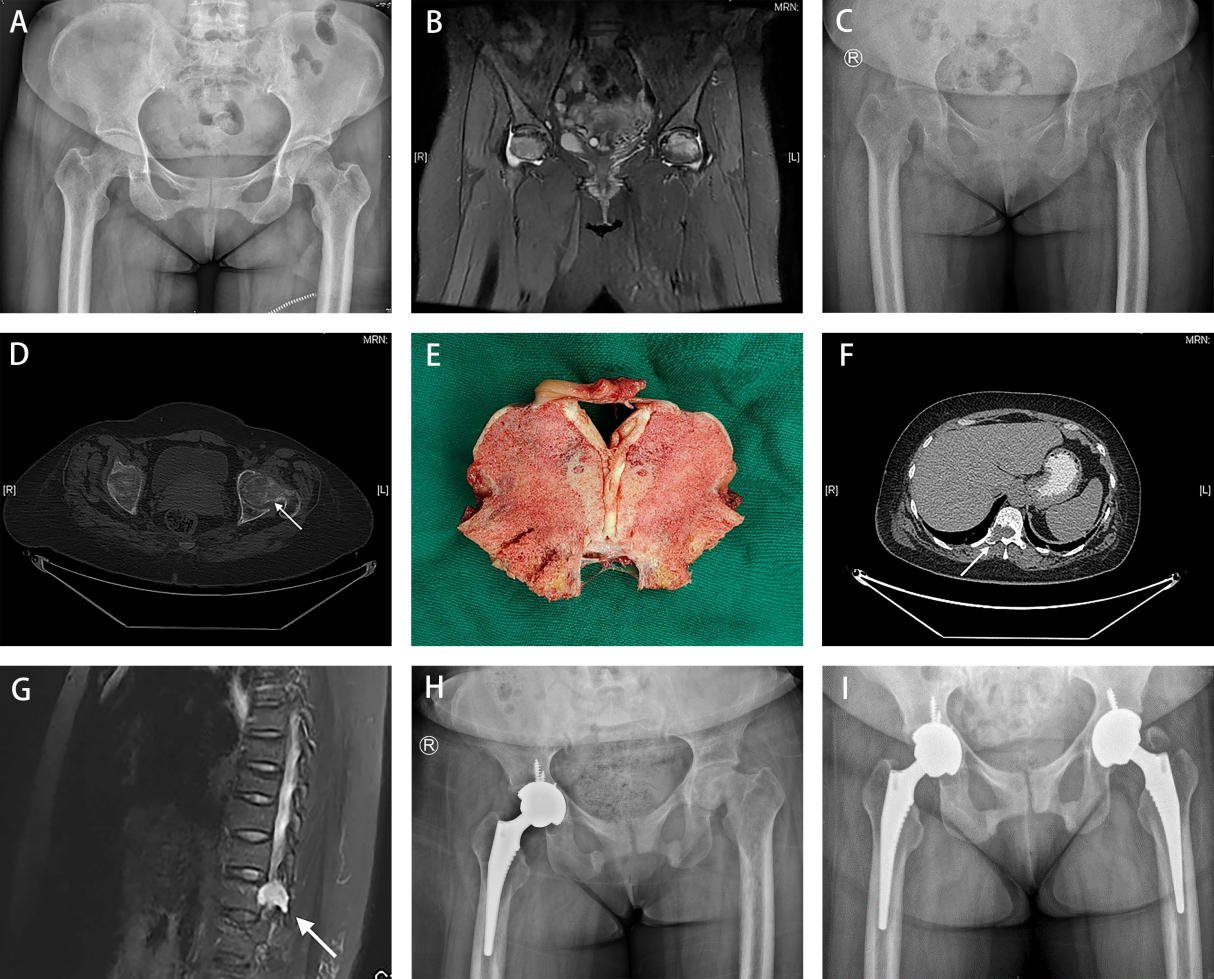
A 49-year-old woman presented consistent bone pain with limb weakness for more than 2 years. **(A)** The pelvic AP radiograph presented grossly normal hips in June 2020. **(B)** Magnetic resonance imaging revealed low-signal intensity bands in the bilateral femoral head. **(C, D)** Images presented blurred bilateral hips with insufficiency femoral neck fracture in June 2022 (indicated by white arrow). **(E)** Severe osteomalacia was found in the femoral head sample. **(F, G)** A well-circumscribed lesion of about 13×18-mm in size was observed in the T11 by PET/CT( white arrow in F) and MRI with long T1 and T2 signals (white arrow in G) in August 2022. **(H)** Radiographs obtained in September 2022 demonstrated the narrow space and coxa vara of the left hip, and proper prosthetic placement without loosening in the right hip. **(I)** Radiographs obtained in March 2023 showed proper bilateral prosthetic positions without signs of loosening.

A 49-year-old perimenopausal woman presented with consistent bone pain with limb weakness persisting for > 2 years. She first sought treatment at our hospital because of bilateral hip pain in June 2020, with normal hip radiograph findings (**Figure 1A**), although low signal intensity were noted in the bilateral femoral heads on magnetic resonance imaging (**Figure 1B**). She was initially diagnosed with early osteonecrosis of the femoral head and underwent nonsurgical treatment. Two years later, from 2020 to 2022, pain extended to the bilateral shoulders and knees with increased intensity; muscle strength of the extremities gradually decreased. The patient was admitted to our hospital for a comprehensive evaluation on 24 June 2022. She had no positive family history or other genetic diseases and her menstrual cycles were regular. Physical examination revealed tenderness of the midpoints of the bilateral groin and restricted bilateral hip range of motion, with grade 3/5 muscle strength in both lower extremities.

All procedures performed in this study adhered to the ethical standards of the institutional and/or national research committee. Written informed consent was obtained from the patient for the publication of this case report.

The results at admission included moderate anemia (hemoglobin 66 g/L), leukopenia (2.70 10^9^/L), neutropenia (1.28 10^9^/L), hypophosphatemia (0.36 mmol/L), high alkaline phosphatase activity (308.00 U/L), and normal serum calcium (2.22 mmol/L). Regarding the evaluation of anemia, low levels of ferritin (4.68 μg/L) were observed, indicating iron deficiency anemia (IDA). Urine phosphorus at 24 h was 0.34 g/24 h (reference range: 0.7–1.7 g/24 h). In terms of bone metabolism markers, the parathyroid hormone levels were 55.90 pg/mL (reference range: 15–65 pg/mL), 25-dihydroxyvitamin D levels (25-OH Vit D) was 11.20 ng/mL (deficiency: < 20 ng/mL), total N terminal procollagen I peptide was 113.00 ng/mL (reference range, premenopausal: 8.53–64.32  ng/mL, postmenopausal: 21.32–112.80 ng/mL), β-Cross Laps was 0.72 ng/mL (reference range: premenopausal 0.068–0.680 ng/mL, postmenopausal 0.131–0.900 ng/mL), and osteocalcin was 22.40 ng/mL (reference range, premenopausal: 4.11–21.87 ng/mL, postmenopausal 8.87–29.05 ng/mL) (**Table 1**). No obvious abnormalities in terms of lung cancer or tumor markers were found. Plain pelvic radiograph obtained in June 2022 showed bilateral hip blur with insufficient femoral neck fractures (**Figure 1C**), and bilateral hip transverse computed tomography (CT) confirmed bilateral femoral neck fractures (**Figure 1D**). Whole-body bone scintigraphy showed radionuclide concentration in the joints without any significantly apparent hot spots in the extremities and spine. Bone mass density (BMD) demonstrated severe osteoporosis in the lumbar spine (0.654 g/cm^2^, T-score -3.8) and the left femur (0.223 g/cm^2^, T-score -5.9).

**Table 1:** Reference ranges for all biochemical data.

Biochemical data	reference range
Hemoglobin	115-150 g/L
Leukocyte	3.5-9.5 g/L
Neutrophil	1.8-6.3 g/L
Blood phosphate	0.85-1.51 mmol/L
Alkaline phosphatase	35-100 U/L
Blood calcium	2.11-2.52 mmol/L
Ferritin	13-150 μg/L
24-hour urine phosphorus	0.7–1.7 g/24 h
Parathyroid hormone	15–65 pg/mL
25-OH Vit D	deficiency: < 20 ng/mL
Total N terminal procollagen I peptide	premenopausal:8.53–64.32 ng/mL, postmenopausal: 21.32–112.80 ng/mL
β-Cross Laps	premenopausal 0.068–0.680 ng/mL, postmenopausal 0.131–0.900 ng/mL
Osteocalcin	premenopausal: 4.11–21.87 ng/mL, postmenopausal 8.87–29.05 ng/mL

Based on these characteristics, the patient was diagnosed with bilateral osteoporotic femoral neck fractures secondary to hypophosphatemia. To alleviate the symptoms of severe pain, the patient was treated with a right THA in July 2022. Severe osteomalacia of the acetabulum and femoral head was observed intraoperatively, with a variation in the shape of the cortical bone as the external force was applied, whereas a nearly normal trabecular appearance was observed despite the lack of rigidity (**Figure 1E**).

During the week following the surgery, additional examinations were performed to explore the cause of hypophosphatemic osteomalacia. Based on the patient’s hematological changes and clinical presentation, we first considered myeloma and examined biochemical markers, including serum immunoglobulin, β2-microglobulin, immunoglobulin light chain, and immunoglobulin electrophoresis. However, all these laboratory findings were within normal ranges and therefore, the suspected diagnosis of multiple myeloma was excluded. Moreover, ^18^F-FDG PET/CT was conducted as a supplementary search, and an osteolytic bone lesion with increased radioactivity in the right side of the T11 vertebral body and adnexa was found, which proved to be a well-circumscribed lesion of approximately 13×18 mm in the T11 with long T1 and T2 signals and an SUVmax of 5.5. on MRI (**Figures 1F, G**).

Subsequently, the patient underwent a CT-guided core biopsy of the lesion, which showed short spindle cells arranged in a hemangiopericytoma-like pattern with no significant cytologic atypia or increased mitotic activity. A panel of immunohistochemistry markers was tested, including CD56, CD34, ERG, SATB2, SSTR2, β-Catenin, S-100, STAT6, SMA, and Ki67. Tumor cells showed positive immunoreactivity to ERG, CD56, SATB2, SSTR2, and β-catenin, but were negative for CD34, S-100, STAT6, and SMA. Furthermore, the Ki67 test showed a very low proliferative index (3%). Based on the morphological characteristics and the immunoprofile described above, the patient was definitively diagnosed with phosphaturic mesenchymal TIO.

After the definitive diagnosis, the patient underwent tumor resection via T11 laminectomy on 6 August 2022. The pathology and immunohistochemistry of the resected tumor were consistent with the findings of the needle biopsy. Bone metabolic markers improved significantly, including serum phosphorus levels increasing from 0.42 mmol/L at 3 days preoperatively to 0.94 mmol/L at the first postoperative evaluation, and alkaline phosphatase decreased from 321.00 U/L to 229.00 U/L, accordingly. Furthermore, the patient experienced large-scale relief from symptoms of bone pain and weakness 3 months after tumor resection. However, she continued to experience persistent pain and limited mobility of the left hip, with a narrow space and coxa vara deformity of the left hip was revealed on radiography (**Figure 1H**). The patient underwent a left-sided THA to restore original function and to reduce physical discomfort and pain. Intraoperatively, we found that bone quality had improved considerably compared to the right side in primary THA, which was consistent with the findings of the preoperative laboratory tests, including hemoglobin 89 g/L, white blood cell counts 3.06 10^9^ cells/L, neutrophil counts 1.66 10^9^ cells/L, ferritin 14.80 μg/L, serum phosphorus 1.29 mmol/L, alkaline phosphatase 168.00 U/L, parathyroid hormone 63.90 pg/mL, 25-OH vitamin D 9.70 ng/mL, total procollagen I N terminal peptide 372.00 ng/mL, β-CrossLaps 3.25 ng/mL, and N-MID osteocalcin of 99.00 ng/mL. The patient’s bone density was reassessed in November 2023. The results showed a lumbar BMD of -1.3 in T score, and with other parts within the normal range of T scores. A six-month follow-up was performed after the second THA. By May 2023, the patient returned to normal gait with satisfactory hip movement function without recurrence of PMT-associated osteomalacia or loosening of the prosthesis (**Figure 1I**).

## 3 Discussion

We report a case of bilateral osteoporotic femoral neck fractures secondary to a PMT at the T11 level, with the following main characteristics: (1) hypophosphatemia and hypophosphaturia, (2) significant anemia and neutropenia, (3) combined bone pain and muscle weakness, (4) marked relief of the aforementioned clinical presentations was achieved after a PMT was detected in the thoracic spine and fully resected, and (5) bilateral osteoporotic femoral neck fractures were successfully treated by THA sequentially.

PMT can occur in almost any soft or osseous tissue. In soft tissues, PMT most often involves the extremities, while bone tumors usually involve the appendicular bones, skull, and sinuses (1). Cases of PMT were reviewed and summarized in 2020 by Garg et al. (5). Since its discovery in 1947, only 21 cases of PMT have been reported involving the spinal column, of which only six were PMT involving the thoracic spine. An analysis of 144 cases of PMT revealed that the most common signs and symptoms of the disease, such as bone pain, difficulty walking, muscle weakness, pathological fractures, and height loss (6), were usually the result of chronic hypophosphatemia rather than a direct consequence of the tumor itself. Our patient had a hip deformity caused by repeated stress fractures in the femoral neck, which left her with symptoms of hip pain even after the complete removal of the primary tumor.

The diagnosis of PMT may be challenging because these tumors are usually small, and it is difficult to identify lesions using imaging technology. The average time from the onset to a correct diagnosis was 2.9 ± 2.3 years (6). In our case, bone scintigraphy revealed active joint inflammation without tumor localization. PMTs commonly express somatostatin receptors (SSTR), as determined by somatostatin receptor scintigraphy using a radiolabeled somatostatin analogue, octreotide (7). However, based on a study by Jadhav (8), ^99^Tc-HYNIC-TOC SPECT/CT and ^68^Ga-DOTA-TATE PET/CT performed equally well and were superior to ^18^F-FDG PET/CT in tumor localization among somatostatin receptor-based scans. ^99^Tc-HYNIC-TOC SPECT/CT or ^68^Ga-DOTA-TATE PET/CT were not performed in this case due to equipment limitations.

Biochemical findings play a crucial role in diagnosis. Patients with PMTs usually demonstrate low serum phosphate, normal serum calcium, elevated alkaline phosphatase, normal or elevated PTH and high serum FGF-23 concentrations. Furthermore, urine phosphate level is an important reference indicator for the diagnosis of TIO. Interestingly, it is high in normal subjects, whereas it was low in this case. Folpe et al. (9) advanced the novel concept of histologically identical tumors not accompanied by phosphaturia or TIO, which they called non-phosphaturic variants. They suggested that these variants may be tumors that secrete inactive or insufficient FGF23. In some reported cases of non-phosphaturic variants (10–12), the patients did not show clinical signs of TIO. In this case, the patient presents only non-phosphaturia, but with clinical and laboratory evidence of TIO. Therefore, we believe that this case was not a non-phosphaturic variant. Nonetheless, the reason for non-phosphaturia-associated TIO is not well understood. The physiological balance of phosphate is maintained by coordinated interactions between the small intestine, bone, parathyroid gland, and kidneys (3). We believe that the most likely reason is that the patient’s phosphaturia level was high at the early stage of the disease and that FGF23 inhibited 25-OH vitamin D synthesis, which in turn affected the intestinal absorption of phosphorus. The prolonged imbalance in phosphorus metabolism resulted in low phosphorus levels in the patient’s body, and although FGF23 inhibited renal tubular reabsorption of phosphorus, the amount of phosphorus that could be filtered by the kidneys was limited, resulting in low urinary phosphorus levels. In addition, patients may suffer from certain asymptomatic digestive disorders, which can also interfere with phosphorus absorption. However, our patient did not undergo endoscopic biopsy. Furthermore, the spine may contain fewer cells that release FGF23; however, serum FGF23 levels were not measured. There was no evidence that PMT caused IDA or leukopenia and we did not find any other comorbidities that contributed to the changes in the hemogram. The diagnosis of multiple myeloma was excluded based on the evaluation of immunoglobulins. The patient’s hematological parameters improved significantly and ferritin levels returned to normal after surgery. Clinicians should pay attention to hypophosphaturia and anemia to avoid misdiagnosis.

A recent study by Chatterjee et al. (13) demonstrated that PMT presents a unique immunophenotype (SATB2+/ERG+/CD56+/S-100-/STAT6-), that corresponded to our case.

Complete resection is the best treatment for PMT (14). Radial resection of the tumor leads to the rapid normalization of biochemical parameters. After surgery, hypophosphatemia and serum alkaline phosphatase levels were significantly ameliorated. However, at 3-month follow-up after tumor resection, the patient still had persistent pain with limited mobility in the left hip. X-rays (**Figure 1H**) showed femoral head collapse and joint space narrowing. The deformity of the femoral head was unchanged compared to the radiographs before the first THA (**Figure 1C**), although a significant increase in bone density was observed. This may be related to osteonecrosis of the femoral head caused by the long-term fracture on it. Therefore, the patient underwent contralateral hip arthroplasty. A recent retrospective study (15) showed that female sex, spine tumors, bone tissue involvement, malignancy, and low preoperative serum phosphorus levels were risk factors for refractory outcomes. Although this patient had more than one identifiable risk factor, she remained disease-free with no evidence of recurrence.

The limitation of this case lies in the absence of FGF23. Although we considered assessing FGF23 for the diagnosis of TIO, the patient chose not to perform this test owing to financial reasons. Consequently, we were unable to observe the dynamic changes in FGF23 throughout the various disease stages. Moreover, we could not offer a conclusive explanation regarding the potential connection between the hypophosphaturia, anemia and neutropenia with FGF23 level.

In conclusion, our report provides detailed clinical data and a diagnostic and treatment approach for the diagnosis of femoral neck fractures caused by TIO. This report should improve the clinical understanding of these rare PMTs and improve patient management. Clinicians should be aware that the long-term prognosis of these patients requires close monitoring.

## Data availability statement

The original contributions presented in the study are included in the article/supplementary material. Further inquiries can be directed to the corresponding author.

## Ethics statement

Written informed consent was obtained from the individual(s) for the publication of any potentially identifiable images or data included in this article.

## Author contributions

YZ: Writing – original draft. MH: Writing – original draft. CG: Data curation, Writing – review & editing. XY: Data curation, Writing – review & editing. SX: Methodology, Writing – review & editing. HX: Writing – review & editing.
